# Morbidity pattern of traditional Chinese medicine primary care in the Hong Kong population

**DOI:** 10.1038/s41598-017-07538-5

**Published:** 2017-08-08

**Authors:** Wendy Wong, Cindy Lo Kuen Lam, Xiang Zhao Bian, Zhang Jin Zhang, Sze Tuen Ng, Shong Tung

**Affiliations:** 1 School of Chinese Medicine, Hong Kong Institute of Integrative Medicine, Chinese University of Hong Kong, 852 Hong Kong, Hong Kong; 2 Department of Family Medicine and Primary Care, University of Hong Kong, 852 Hong Kong, Hong Kong; 3 Clinical Division, School of Chinese Medicine, Hong Kong Baptist University, 852 Hong Kong, Hong Kong; 4 Hong Kong Bone-Setting Specialist Centre, Modern TCM Limited, 852 Hong Kong, Hong Kong; 5 Hong Kong Registered Chinese Medicine Practitioners Association, 852 Hong Kong, Hong Kong

## Abstract

Primary care manages >90% of illnesses requiring medical services in Hong Kong, in which 9,513 registered Chinese medicine practitioners (CMPs) provide 8.2% of the consultations. This is the first study aimed to determine the morbidity pattern in different Traditional Chinese Medicine (TCM) primary care settings in Chinese population. 55,312 patients’ encounters were classified by the International Classification of Primary Care-2 (ICPC-2) from 260 of CMPs. Mean patient age was 50.5 years, with more females than males (67.0% vs 33.0%). Most patients consulted CMPs for chronic (64% vs 33.7%) rather than acute conditions. Among the 30% of patients, hypertension (49.5%) or diabetes (18.5%) were the most common co-morbidity. The most common problems presenting to CMP were respiratory (24.9%), musculoskeletal complaints (22.7%), cough (11.7%), and lower back pain (6.6%). To our knowledge, this was the first study permitting direct comparison with that presenting to Western medicine (WM) primary care by ICPC-2 systems. The results confirmed the role of CMP in primary care for musculoskeletal or chronic illnesses that they may have also received conventional WM treatment. We recommend greater effort and more resources should be invested to promote interdisciplinary communication to ensure safety and synergy of TCM and WM in primary care.

## Introduction

As an alternative to Western medicine (WM), more patients across the globe are turning to complementary and alternative medicine (CAM), such as traditional Chinese medicine (TCM), for primary care^1^. Primary care manages over 90% of illnesses in Hong Kong, thus providing constant surveillance concerning public health^2–4^. Although WM is considered to be the main choice of care in the global health care system context, the importance of TCM in primary care is starting to be recognised, with increased use in recent years^1^. In 1997, the number of CAM visits exceeded the number of visits to all primary care physicians, with an estimated total out-of-pocket expenditures on CAM of US$27 billion; this was comparable to the expenditures for all primary care physician services for the same year^5^. TCM, including the prescription of Chinese herbs, acupuncture and bone-setting, is one of the most popular CAMs globally and is practised widely in Asia, the United States, Canada, Europe, and Australia^6–8^. TCM comprises up a major proportion of CAM services in the US^7^, increasing from 34% in 1989 to 38.3% in 2007^9^. In Denmark, the proportion of patients who had used TCM at least once annually increased from 23% in 1987 to 43.7% in 2007^10^.

The increasing usage of TCM profoundly affects global health care services. The National Centre of Complementary and Alternative Medicine and the National Health Service have been established in the US and the United Kingdom, respectively, to allocate national budget for TCM services in primary care. Other European countries also have provided public financing for TCM^5^. In addition, the global increase in the use of TCM has initiated calls for more information regarding its function and outcomes to better guide medical resource allocation^10–13^.

In Hong Kong, which has a health care system similar to that of the majority of developed countries, 50–60% of the population consulted a Chinese medical practitioner (CMP) at least once in their lives, and 13.5% consulted CMPs occasionally or frequently^14–16^. Our recent population survey showed that 8.2% of primary consultations were provided by CMPs and that many patients in Hong Kong regarded CMPs as their family doctors^17^. With 9,513 registered CMPs in Hong Kong providing primary care service^18^, the importance of TCM for patients with chronic diseases has been increasing rapidly since the establishment of the Chinese Medicine Ordinance and the regulation of CMPs in 1999^19^. In response to this increasing demand, the management modalities of CMPs are evolving. For example, for added convenience, herbal medicines are available in tablet or powder forms in addition to the traditional raw herbs, and CMPs may refer patients to other specialists for further examination. However, there are wide variations in training and practice settings amongst CMPs in Hong Kong. Information on patient management by CMPs in terms of prescriptions, physical treatments (e.g., Tui-Na, cupping), preventive care, and referrals are useful for quality assurance and training of CMPs. Currently, there are no standardised postgraduate or specialty training requirements for CMPs. Moreover, it is not clear whether the characteristics of CMPs have any impact on morbidity patterns or the patients; such characteristics include undergraduate education; place of training; postgraduate training; years of clinical experience; practice setting, including affiliation with a public hospital, non-governmental organisation, or herbal shops; group or solo practice; or methods of payment.

Morbidity data are important to policy makers and researchers as it can be an indicator of complaints, conditions, and health of the population^20^. However, there is no morbidity pattern research that compares both conventional medicine and TCM by using international coding systems. To establish the platform of interdisciplinary communication, one study mapped diagnoses of functional dyspepsia between TCM and WM^21^, but the sample size was too small to provide an representative inference. Therefore, results of a large morbidity survey from the general population regarding patient encounters by CMPs could supplement those of WM and could provide a more comprehensive view regarding the prevalence of common illnesses amongst the Chinese population^22^. Furthermore, there has been an announcement of the first TCM hospital in Hong Kong by the year 2025 and discussion regarding the role of CMPs in fulfilling a primary role for medical services in an aging population, such a role includes treatment for cancer, post-stroke, and musculoskeletal pain treatment^23^. Accordingly, elucidation of the morbidity pattern of TCM in primary care could provide information on the service gaps and the needs of TCM, allowing for better planning and resource allocation of primary care in Hong Kong.

The need for TCM morbidity research has been emphasised with regards to its importance in the development of health policy^12^, professional development, clinical audit, education, and research. Morbidity surveys of TCM have been carried out in the United Kingdom, Australia, and Switzerland^11, 24, 25^. These studies found that patients consulting TCM practitioners most commonly presented with musculoskeletal disorders and tended to have more chronic and severe illnesses. Our pilot study in the TCM primary care clinic of Tung Wah Hospital in Hong Kong found a different morbidity pattern from that of WM primary care clinics^26^, but a large-scale, population-based morbidity survey is lacking to confirm the role of TCM primary care in Hong Kong.

The current study aimed to determine the overall morbidity pattern of patients from different TCM primary care settings in Hong Kong as well as to analyse morbidities based on age and sex. To the best of our knowledge, this is the first morbidity survey to determine the morbidity pattern of TCM primary care by using both WM and TCM common coding measures. The results of this study can provide useful information for the allocation of resources and changes in service utilisation.

## Methods

### Study design, setting, and patients

This was a cross-sectional study collecting data from all clinical encounters during the first week of every month from January 2012 to February 2013 from different CMP settings and districts in Hong Kong. The sampling frame was targeted at CMPs from TCM clinics affiliated with the Hospital Authority, universities, or registered members of the Hong Kong Chinese Medicine Practitioners Association consisting of 80% of all registered CMPs in Hong Kong. Characteristics of the CMPs are summarised in Table 1. CMPs of this study ranged in age from 24 to 73 years old (mean 46.1), 68.5% of whom were male. Those who had acquired a TCM-related master degree comprised 38.1%, and specialists in Chinese medicine comprised 80.8%. The median number of years in practice was 7 years, and most CMPs (42%) worked in the private sector. The sex ratio and the practice characteristics of CMPs in this study resembles that of the manpower survey conducted by the Hong Kong government^27^.

**Table 1:** Characteristics of Chinese medicine practitioners who participated in the morbidity survey.

CMP (n = 260)	Mean (SD)
Age	46.1 (17.2)
Number of years in general practice	18.3 (17.4)
Number of hours per day	7.5 (1.5)
Number of cases per day	17.4 (9.7)
**Sex**	n (%)
Male	178 (68.5)
Female	82 (31.5)
**District of practice**	
Hong Kong Island	49 (18.8)
Kowloon	56 (21.5)
New Territory	113 (43.5)
Mixed districts	42 (16.2)
**Place of graduation**	
Hong Kong	147 (56.5)
China	108 (41.5)
Both	5 (1.9)
**Type of organisation**	
Government/Hospital Authority	41 (15.8)
Non-government organisation (NGO)	86 (33.1)
University-affiliated clinic	23 (8.8)
Private solo/group	109 (42.0)
Other	1 (0.4)
**Post-graduate training**	
None	59 (22.7)
Certificate	63 (24.2)
Higher diploma	5 (1.9)
Master degree	99 (38.1)
Doctoral degree	34 (13.1)

### Sample size calculation

Sample size calculation was based on the primary objective of determining health problem/diagnosis percentages of all clinical encounters in primary care in Hong Kong. The percentages were conservatively anticipated to be 50%, with a 95% confidence interval and 0.5% maximum error. Based on these numbers, a minimum of 38,415 encounters were needed. Data from 55,312 patients from 260 CMPs were collected over four seasons, which were based on the summary of meteorological observations in Hong Kong in 2009 for monthly temperature and relative humidity as a sampling frame^28^. Therefore, about 10,000 encounters per season were included. In a service utilisation survey conducted by the corresponding author in Hong Kong^26^, each CMP had about 200 encounters per week (average 40 consultations per day); to achieve 10,000 encounters per season, this equates to 50 CMP-weeks. To account for an anticipated dropout rate of 30%, 75 CMPs were required to collect one week of data in each season to be surveyed, taking into consideration the variation in the workload among different CMPs.

### Data collection

A total of 260 CMPs recorded health problems of all clinical encounters during each data collection week. The presenting problem was coded according to the International Classification of Primary Care (ICPC)-2^29^. All completed study forms were returned to the research team after each data collection week. WM doctors coded the presenting complaints into ICPC-2 codes. A double key-in system was used to assure the quality of data entry. All data received were checked for coding, entry, cleaned, and compiled into a central database for analysis. All methods were carried out in compliance with the Institutional Review Board of the University of Hong Kong/ Hospital Authority Hong Kong West Cluster (HKU/HA HKW IRB) and human ethics approval for the current study was granted reference number UW 10-414. Informed consent was obtained from all CMPs for providing patients’ data without any patients’ identifiers. As no data that could be used to identify any patients or care providers for the database construction and informed consent of the patients was unnecessary, this was approved by the HKU/HA HKW IRB before the results were released. Therefore, it is impossible to query the data alone to identify individuals by any means. (http://www.hkuctr.com/Study/Print/7dbba90eaa7e430d99d396f5c473eee5).

### Outcome measures

Primary outcomes were (1) the presenting health problems of all clinical encounters during the study period and (2) prevalence of chronic problems. Secondary outcomes were the associations of morbidity patterns with CMP characteristics and patient sociodemographic data.

### Data analysis

Morbidities were expressed as the percent distribution of different types of ICPC-2-coded health problems and were compared with data from a previous Western medicine morbidity survey^22^. The proportion of chronic health problems and preventive care offered were expressed as the percent distribution. Logistic regression analysis was used to examine the effects of CMP background (age, sex, education background, qualification, or postgraduate training) and practice characteristics (proportion of chronic diseases encountered) on morbidity patterns. Independent variables were classified based on (1) patient demographics; (2) CMP practice characteristics, including affiliation and payment methods (out-of-pocket, insurance, employer); and (3) characteristics of CMP including age, sex, years of experience, place of TCM education, undergraduate degree in TCM, postgraduate training, and postgraduate qualifications. For each regression analysis, an unadjusted analysis was first performed followed by an analysis adjusted for health problems, demographics, and chronic morbidity. We analysed the data using SPSS 20.0, and a 95% confidence interval for each estimate and a 5% level of significance in all statistical tests.

### Ethics

Ethics approval for the current study was granted by the HKU/HA HKW IRB (UW 10-414).

## Results

### CMP and patient characteristics

The demographic characteristics of 260 CMPs and 55,312 of patients are shown in Tables 1 and 2, respectively. The mean age of the study sample was 50.5 years, which is higher than that of the general Hong Kong population (41.0 years) and patients who consulted a WM outpatient clinic (43.5 years)^22, 30^. The ratio of female to male patients (2:1) was also higher in the current study sample compared to that in the general Hong Kong population (1.2:1)^30^. Patients aged 45–64 years (36.6–38.3%) or who were female were more likely to consult CMPs in both private and public primary care settings. The majority (64%) of patients consulted CMPs for chronic illnesses; in contrast, 36% consulted WM practitioners for chronic illnesses^22^. Regarding form of payment, more than 84% of patients consulting CMP paid out-of-pocket in both public and private sectors; this was more than that in WM primary care (62%). It should be noted that the patient load of the public sector (22.2) was much higher than in the private sector (12.1), considering a similar number of daily working hours for CMPs.

**Table 2:** Characteristics of patients under care of Chinese medicine practitioners in the private and public sectors.

	Public sector (n = 29,763)	Private sector (n = 25,340)	TCM Total (n = 55,103)	General population (2013) (n = 71,875,000)	WM (n = 52,337)
*Age Mean (SD) (years)	52.6 (19.7)	48.0 (19.4)	50.5 (19.7)	41.0 (20.9)	43.5 (21.9)
Range	0–100	0–101	0–101	0–100+	0 −
Female-to-male ratio	1.97:1	2.09:1	2.01:1	1.2:1	1.4:1
^†^Age group (years), n (%)	n (%)				
<15	1312 (4.4)	1241 (4.9)	2562 (4.6)	(11.1)	NA
15–24	1450 (4.9)	1685 (6.6)	3156 (5.7)	(12.0)	
25–44	6609 (22.2)	7861(31.0)	14510 (26.2)	(31.1)	
45–64	11414 (38.3)	9276 (36.6)	20772 (37.6)	(31.7)	
>65	8978 (30.2)	5277 (20.8)	14303 (25.9)	(14.2)	
*Nature of health problem					
Acute	8387 (28.2)	10071 (39.7)	18626 (33.7)	NA	41166 (58.9)
Chronic	20779 (69.8)	14611 (57.6)	35415 (64)		25045 (35.8)
Preventive	600 (2.0)	664 (2.6)	1271 (2.3)		3693 (5.2)
^†^Payment method					
Self-payment	23259 (78.1)	22831 (90.1)	46258 (83.6)		32340 (62.0)
Government subsidy	2601 (8.7)	11 (0)	2612 (4.7)		3258 (6.2)
Medical insurance	1093 (3.7)	2069 (8.2)	3180 (5.7)		61124 (30.9)
Health care voucher	822 (2.8)	283 (1.1)	1119 (2.0)		NA
Others	1991 (6.7)	152 (0.6)	2143 (3.9)		406 (0.8)
Number of CMPs	150	110	260		
*Number of hours per day	7.44 (1.3)	7.93 (1.4)	7.67 (1.34)		8.0 (1.3)
*Number of patients per day	22.23 (7.8)	12.13 (8.7)	17.56 (9.64)		NA

TCM, traditional Chinese medicine; WM, Western medicine; SD, standard deviation; CMP, Chinese medicine practitioners; NA, not available. Note: *p < 0.05 between public vs private sectors; TCM vs WM samples by independent sample t-test or chi-square test. ^†^p < 0.05 between public and private sectors by independent sample t-test or chi-square test.

### Morbidities under CMP and WM care were different

Table 3 shows all health encounters recorded by the CMPs and compares it with the 2007 Hong Kong WM morbidity survey^22^. The 30 most common primary care symptoms comprised 75% of all health encounters with CMPs in Hong Kong. Based on ICPC-2 coding, the most common health problems encountered by CMPs fell under the categories of respiratory (R, 24.9%), musculoskeletal (L, 22.7%), neurological (N, 11%) and skin (S, 5.6%). The specific top three health problems were cough (R05, 11.7%), low back symptoms/complaints (L03, 6.6%), and sleep disturbance (P06, 3.8%), comprising 22.2% of all health problems. Compared to problems encountered at WM clinics, the following health problems were more frequently encountered by CMPs: general (A) (6.1% vs 4.5%), musculoskeletal (L) (22.7% vs 7.1%), neurological (N) (11.9% vs 1.6%), and female genital (X) (4% vs 2.1%). The most common general conditions encountered by CMPs were weakness (A04, 1.2%), chills (A02, 1.2%), and health main/preventive medicine (A98, 1%). Musculoskeletal complaints including problems with the low back (L03, 6.6%), knee (L15, 3%), neck (L01 2.9%), shoulder (L08, 2.4%), foot (L17, 2%), thigh (L14, 2%), and ankle (L16, 1.2%). Neurological complaints consisted of dizziness (N17, 3.2%), paralysis (N18, 2.5%), and neurological disease (1.4%). Female genital complaints consisted primarily of scanty or absent menstruation (X05, 0.9%).

**Table 3:** Health problems encountered in Chinese and Western medicine^22^ settings.

Health Problem (ICPC-2)	*Patient encounters with Chinese medicine practitioners (n = 55,312)	*Patient encounters with Western medicine practitioners (n = 68,140)
	n (%)	
General (A)	3361 (6.1%)	3085 (4.5%)
Blood (B)	42 (0.1%)	336 (0.5%)
Digestive (D)	5144 (9.3%)	6702 (9.8%)
Eye (F)	291 (0.5%)	1380 (2.0%)
Ear (H)	403 (0.7%)	725 (1.1%)
Cardiovascular (K)	1363 (2.5%)	8756 (12.8%)
Musculoskeletal (L)	12,576 (22.7%)	4839 (7.1%)
Neurological (N)	6603 (11.9%)	1067 (1.6%)
Psychological (P)	2795 (5.1%)	1766 (2.6%)
Respiratory (R)	13,792 (24.9%)	24,653 (36.2%)
Skin (S)	4646 (8.4%)	5049 (7.4%)
Endocrine (T)	615 (1.1%)	6360 (9.3%)
Urological (U)	866 (1.6%)	943 (1.4%)
Reproductive (W)	496 (0.9%)	374 (0.5%)
Female genital (X)	2188 (4.0%)	1431 (2.1%)
Male genital (Y)	127 (0.2%)	554 (0.8%)
Social problems (Z)	4 (0.0%)	118 (0.2%)

^*^Statistical significance difference between patient encounters in Chinese and Western medicine settings by chi-squared test (p < 0.05).

### Pre-existing morbidities commonly found in CMP care

Table 4 shows that 30.3% of patients consulting CMPs possessed at least one other prior chronic comorbidity. The three most common pre-existing chronic diseases were hypertension (K86, 49.5%), diabetes (T90, 18.5%), and allergic rhinitis (R97, 6.1%). Among paediatric (<15 years) and adolescent (15–24 years) patients, the most common disease was allergic rhinitis at 63.2% and 45.2%, respectively, while among young adult (25–44 years) patients, the most common was depressive disorder at 10.6%. In both adult and elderly patients, the most common were hypertension (40.5% and 67.1%, respectively) and diabetes (17.3% and 23.5%, respectively). More females patients had depressive disorders (5.6%), while more males patients had stroke/cerebrovascular accidents (6.1%). In addition, more male patients with hypertension (52.2%) were managed by CMPs.

**Table 4:** Co-morbidities of the five most common chronic conditions based on ICPC-2 coding, separated by age group and sex.

Chronic morbidity		Overall	Age group					Sex	
			Paediatrics < 15	Adolescents 15–24	Young adult 25–44	Adults 45–64	Elderly ≥ 65	Male	Female
Size (n)		45134	2186	2473	11665	16973	11828	14800	30315
Chronic diseases (n, (%))		13686 (30.3)	291 (13.3)	228 (9.2)	1371 (11.8)	4848 (28.6)	6944 (58.7)	4877 (33.0)	8803 (29.0)
		**(Column %)**							
K86	Hypertension uncomplicated	49.5	0.34	0.88	11.31	40.5	67.1	52.2	48.1
T90	Diabetes non-insulin dependent	18.5	0.00	0.00	4.2	17.2	23.5	20.0	17.7
R97	Allergic rhinitis	6.1	63.2	45.2	17.4	5.1	0.9	6.8	5.7
P76	Depressive disorder	4.2	0.0	0.4	10.6	6.7	1.5	1.6	5.6
K90	Stroke/cerebrovascular accident	3.7	0.0	0.0	0.0	3.1	5.0	6.1	2.3

### Different complaints reported by different age groups consulting CMPs compared to those consulting WM doctors

Table 5 compares common health problems by age group encountered by CMPs with those encountered by WM practitioners. In general, respiratory complaints were the most common across different age groups (9.3–23.9%), followed by skin problems (acne, 9.6%) in adolescents and musculoskeletal complaints (low back complaints) in adults and the elderly (7.1% and 8.6%, respectively). Our morbidity survey shows that CMPs managed more patients with allergic rhinitis (4.1–10.6%), paediatric patients with allergic contact dermatitis (3.5%), and adolescent patients with acne (9.6%). In addition, young adult and adult patients were more likely to visit a CMP for treatment for sleep disturbance (4.4–4.6%), as were young adult patients with scanty or absent menstruation (2.6%). For the elderly, more elderly patients reporting vertigo/ dizziness (5.1%) were managed by CMPs.

**Table 5:** Health problems encountered in Chinese and Western medicine^22^ settings across different age groups.

(%)	Paediatrics		Adolescents		Young adult		Adults		Elderly	
	CM n = 2562	WM n = 4989	CM n = 3156	WM n = 3619	CM n = 14510	WM n = 23,393	CM n = 20,772	WM n = 20,913	CM n = 14,303	WM n = 15,082
1	Cough (23.9)	Upper respiratory tract illness (52.1)	Acne (9.6)	Upper respiratory tract illness (41.9)	Cough (9.3)	Upper respiratory tract illness (34.9)	Cough (10.8)	Upper respiratory tract illness (19.4)	Cough (13.9)	Hypertension (23.7)
2	Sneezing/nasal congestion (14.6)	Immunisation, acute bronchitis (6.0)	Cough (9.3)	Gastroenteritis (7.6)	Low back symptom/ complaint (6.1)	Gastroenteritis (6.0)	Low back symptoms/ complaints (7.1)	Hypertension (13.9)	Low back symptoms/ complaints (8.6)	Upper respiratory tract illness (10.8)
3	Allergic rhinitis (10.6)	Gastroenteritis (4.8)	Sneezing/nasal congestion (7.1)	Dermatitis (4.1)	Sneezing/nasal congestion (4.5)	Dermatitis (3.2)	Sleep disturbance (4.6)	Diabetes (6.0)	Vertigo/ dizziness (5.1)	Diabetes (8.9)
4	Rash localised (4.4)	Dermatitis (4.2)	Rash localised (6.3)	Allergic rhinitis (3.3)	Sleep disturbance (4.4)	Physical check-up (2.7)	Headache (4.5)	Lipid disorder (4.5)	Knee symptoms/ complaints (5.1)	Lipid disorder (5.0)
5	Dermatitis contact/allergic (3.5)	Influenza (2.6)	Throat symptoms (4.5)	Acute bronchitis (3.1)	Neck symptoms/ complaints (4.1)	Acute bronchitis (2.3)	Knee symptom/ complaint (3.5)	Gastroenteritis (2.5)	Paralysis/ weakness (4.6)	Osteoarthritis, cerebrovascular disease (2.4)
6	Rash generalised (3.4)	Allergic rhinitis (2.1)	Allergic rhinitis (4.1)	Influenza (3.0)	Headache (4.0)	Immunisation (2.0)	Neck symptom/ complaint (3.4)	Acute bronchitis, dermatitis (2.1)	Headache (3.4)	Dermatitis (1.9)
7	Fever (3.1)	Asthma (1.6)	Ankle symptoms/ complaint (3.5)	Immunisation (2.5)	Throat symptom/ complaint (3.9)	Allergic rhinitis (1.6)	Shoulder symptom/complaint (3.4)	Physical check-up (1.9)	Neurological disease other (3.4)	Gout (1.8)
8	Pruritus (2.7)	Acute tonsillitis, infectious conjunctivitis (1.3)	Pruritus (3.0)	Acne (2.1)	Allergic rhinitis (3.4)	Abdominal pain (1.5)	Throat symptoms (3.3)	Dyspepsia (1.5)	Sleep disturbance (3.0)	Ischemic heart disease (1.7)
9	Psychological disorders (2.3)	Acute otitis media, skin infection, chickenpox, abdominal pain, cough (0.7)	Headache (2.7)	Acute tonsillitis (1.6)	Rash localised (2.8)	Influenza, hypertension (1.4)	Vertigo/dizziness (3.2)	Immunisation, abdominal pain (1.2)	Swollen ankles/oedema (2.4)	Acute bronchitis (1.6)
10	Throat symptoms (2.3)	Blepharitis/stye/ chalazion, other viral rash/disease, urticaria (0.6)	Sleep disturbance (2.7)	Abdominal pain (1.5)	Menstruation absent/ scanty (2.6)	Dyspepsia, acute tonsillitis (1.2)	Sneezing/nasal Congestion (2.8)	Anxiety, test results (1.1)	Shoulder symptoms/complaint (2.2)	Benign prostatic hypertrophy (1.4)

TCM, traditional Chinese medicine; WM, Western medicine.

### Effects of CMP characteristics on primary care morbidity patterns

Of the surveyed CMPs, 57.2% held a bachelor degree or below, while 51.2% had a master degree or higher (Table 1). After controlling for sociodemographic data of the CMP, postgraduate or higher qualifications of CMP were associated with a higher proportion of consultations for chronic problems (71.7%; p < 0.001). The proportion of consultations associated with chronic diseases for CMPs affiliated with public hospitals was 40.4%, compared to 16.9% for CMPs affiliated with private organisations (p < 0.001). No other factors were found to relate to the trend of the morbidity pattern.

## Discussion

### Significance of the current study

This study highlights the importance of TCM in primary care in Hong Kong by categorising health conditions presented to CMPs according to the ICPC-2 classification system; this allows direct comparison with previous morbidity patterns presenting to practitioners of WM. This provided a better understanding regarding how TCM complements WM based on empirical data obtained in the medical records. As CMPs are often the first medical professionals that patients approach in Chinese cultures, the morbidity pattern associated with TCM should be considered for developing effective health measures in primary care. With the global concern in the use of integrative medicine in medical systems, this study helps to illustrate how the overall practice of primary care can be improved and better utilised in Hong Kong or other settings.

### Sample characteristics

The study sample included more elderly and female patients when compared with the Hong Kong general population in 2013, reflecting that these groups of patients were more likely to seek TCM primary care, which was consistent with other studies^26, 31^. In addition, patients were more likely to consult CMPs for chronic illnesses, thus confirming the role of CMPs in primary care for chronic disease management. On the other hand, the majority of the Chinese population will opt for Western allopathic medicine over TCM for acute illnesses^3^. In contrast, for chronic illnesses for which conventional medicine tends to have a low treatment efficacy, patients tend to prefer TCM for treatment^3^. The Hong Kong Special Administrative Region government provides tripartite (i.e. Hospital Authority, non-governmental organisation, and university) public TCM outpatient services; however, more than 84% of patients pay for TCM services out-of-pocket, reflecting that the financial burden borne by the patients and the demand have been underestimated. Although the emerging evidences from systematic reviews or meta-analyses confirmed the role of TCM in chronic diseases management in cancers, cardiovascular disease or asthma^7, 32, 33^, TCM is still not incorporated into conventional medical systems in countries other than China. Reinforcement of regulation and training of CMPs in Asia has formally been upheld at the university level, thus ensuring the coordination and continuity of TCM in Chinese populations^32^. By providing equal access to primary care through either TCM or WM, the incorporation of TCM in conventional medicine should be increased to facilitate better quality of care of aging populations^7, 34^. The current study illustrates that CMP is an important vehicle through which to promote primary care services. Notwithstanding, without a clear TCM or WM referral platform in Hong Kong for further clinical follow-up, the management of advanced medical condition of patients could place patients at risk and result in the medical system not being optimised^8, 35^. To facilitate interdisciplinary mutual understanding (i.e. between TCM and WM), established official regulations are needed to enhance patient care^36^.

### Importance of ICPC-2 for translational research of TCM

Diagnostic classification systems such as the International Classification of Disease (ICD) or International Classification of Primary Care (ICPC) are used in most primary care studies to classify the results^13, 29, 37^. Accordingly, to create a more comprehensive view of health care service utilisation in Hong Kong, ICPC-2 coding of health problems in the TCM context of the current study is crucial for direct comparisons with these studies, such as the 2007–2008 morbidity survey of WM^22^. In fact, TCM treats syndromes/patterns that are typically described in terms that are more traditional. In facilitating communication, these syndromes are currently associated to or terms from a biomedical understanding and were found to be very crucial for effective personalised medicine^38^. Past morbidity surveys lack of information from the TCM point-of-view that could be useful for CMP practice^12^. If standardised classification of TCM diagnoses becomes common practice, this will enable collection and comparison of these data across studies, years, and countries. This study provides additive data regarding primary care from both sectors and will aid in providing better projections of the needs in the overall primary care system.

### Targeted planning of primary care in Hong Kong

As shown in Table 3, the morbidity pattern of TCM differs from that of WM^22^. Although respiratory problems ranked the highest in both TCM and WM, the proportion of respiratory problems in the TCM sample was 10% lower than that reported for WM. Moreover, the proportion of musculoskeletal problems encountered by CMPs was approximately 15% higher than in WM and was the second highest proportion of the health problems in TCM primary care services. In addition, consultation demands for general, neurological, and female genital problems were never emphasised. Although clinical trials demonstrated the effectiveness of Chinese herbal medicine or acupuncture with regards to the common cold^39^, stroke^40^, insomnia^41^, or pain management^42, 43^, it is unclear whether the qualifications of Hong Kong CMPs had equipped them to provide the best medical care to these patients. A higher standard of medical education or official specialty training are needed to uphold the quality of care to meet the demands of the morbidity pattern reported herein. With the increasing burden of the aging Hong Kong population, the prevalence of chronic conditions in terms of musculoskeletal and neurological problems will also increase. The different distributions of health problems between TCM and WM indicate that targeted planning of primary care in Hong Kong is essential to improve the quality of care, a notion supported by other groups^44^. Although our study showed a population demand on TCM, the underlying disease may not have sufficient evidence^45, 46^. Echoed with the evidence based medicine for Traditional Medicine (TM)^7, 47^, health policy makers should target and allocate resources on the disease areas that are in accordance to the CONSORT extension guidelines on TCM^48^. With the Hong Kong Government dedicated to develop evidence based TCM^8, 23, 49^, the Hong Kong hospital authority has invested on pilot programmes on cancer palliative care, stroke rehabilitation and chronic low back pain^8^. With sophisitcated data provided in this study which has never been investigated, resources should be extended to areas with the best evidence support so as to improve quality of care and safety of our primary care.

### Integration of TCM in primary care in Hong Kong

Future development of integrated primary care should consider the TCM patient base and disease profile^50^. Notably, based on the morbidity pattern described herein, TCM plays a major role in providing complementary primary care services apart from WM. To prepare for the needs of an aging Hong Kong population^30^, these results suggest that future development of primary care should emphasise treating chronic health problems. Furthermore, symptom-specific treatments should be developed and targeted for the needs of different age groups and sexes. In particular, the current results illustrate that allergic rhinitis and dermatitis care should be targeted for paediatric patients or their respective care givers, as more than half of this group (56.4%) experienced these problems (Table 5). Similarly, care for elderly patients should focus on musculoskeletal and neurological conditions, care for teenage patients should focus on skin care, and care for female teenage patients should focus on female genital and neurological care. The successful campaign of therapeutic acupuncture to treat adults with psychological distress is a good example of how TCM can help relieve the workload of WM practitioners^51^. Developing TCM-related campaigns would also likely enhance the overall knowledge of patients and their ability to communicate concurrent CAM therapies with their doctors, thereby avoiding potential misunderstandings and improving the overall health outcome. This idea has been supported by studies from other countries^52, 53^.

### CMP practice characteristics and implications

As expected, Chinese herbs were the most common form of prescription in TCM. However, Chinese powders have a growing importance in TCM due to their convenience and because they can be more easily controlled for quality and dosage. In addition to physical treatments provided for the patients, CMPs also often provide lifestyle advice. A previous study found that lifestyle advice provided by CMPs, focusing on diet, exercise, and stress management, differs from that provided by WM practitioners, focusing on smoking and alcohol^54^. This again highlights the importance of CMPs in providing primary care to the Hong Kong population. The current study shows that private sector CMPs and those with more experience are more willing to give lifestyle advice and spend additional time with each patient, perhaps to maintain a better doctor-patient relationship. With the scope of chronic disease management of diabetes as well as hypertension of the aging population, the current results show that CMPs’ years of clinical experience, level of education, and hours of practice per day affect the type of treatment prescribed; further studies should fully elucidate these relationships in terms of patients’ enablement or empowerment in primary care^55^.

## Conclusions

This was the first study to investigate morbidity patterns of CMP patients in Hong Kong using ICPC-2 coding in the primary care setting. This system of classification is effective for this patient group, as at least 75% of all health problems were captured by the 30 most common ICPC-2 classifications. Patients in TCM settings consulted mostly regarding chronic health problems, which is also true in WM settings. However, the morbidity pattern of TCM settings was different from that of WM, with more consultations on musculoskeletal, neurological, and psychological problems, but less on respiratory, cardiovascular, and endocrine problems in TCM than in WM. Standardised classification of diagnoses from CMPs will allow continued comparisons of morbidity patterns between TCM and WM over time, allowing for better understanding and planning of primary care.

This study also revealed key information on the management patterns of CMPs. Chinese herbs were the most common form of treatment in TCM primary care, and the use of Chinese powders as a replacement for Chinese herbs is growing. The difference in care from CMPs from the public versus private sectors and from younger versus more experienced CMPs was also elucidated. Further studies should be conducted on the practice environments, education background, and management patterns of CMPs in Hong Kong to help standardise and optimise the quality of primary care received from CMPs across Hong Kong.
